# An intracellular motif of GLUT4 regulates fusion of GLUT4-containing vesicles

**DOI:** 10.1186/1471-2121-9-25

**Published:** 2008-05-20

**Authors:** Catherine A Heyward, Trevor R Pettitt, Sophie E Leney, Gavin I Welsh, Jeremy M Tavaré, Michael JO Wakelam

**Affiliations:** 1 CR-UK Institute for Cancer Studies, Birmingham University, Birmingham B15 2TT, UK; 2 Department of Biochemistry, University of Bristol, Bristol BS8 1TD, UK; 3 Babraham Institute, Babraham Research Campus, Cambridge, CB22 3AT, UK

## Abstract

**Background:**

Insulin stimulates glucose uptake by adipocytes through increasing translocation of the glucose transporter GLUT4 from an intracellular compartment to the plasma membrane. Fusion of GLUT4-containing vesicles at the cell surface is thought to involve phospholipase D activity, generating the signalling lipid phosphatidic acid, although the mechanism of action is not yet clear.

**Results:**

Here we report the identification of a putative phosphatidic acid-binding motif in a GLUT4 intracellular loop. Mutation of this motif causes a decrease in the insulin-induced exposure of GLUT4 at the cell surface of 3T3-L1 adipocytes via an effect on vesicle fusion.

**Conclusion:**

The potential phosphatidic acid-binding motif identified in this study is unique to GLUT4 among the sugar transporters, therefore this motif may provide a unique mechanism for regulating insulin-induced translocation by phospholipase D signalling.

## Background

The lipid second messenger, phosphatidic acid (PtdOH) is produced by phospholipase D (PLD) in a variety of intracellular signalling pathways, including cell adhesion and migration, vesicular trafficking and phagocytosis [1-3]. Phosphatidic acid transduces the signal by altering the localisation and/or activity of its target proteins. A number of proteins are known to be regulated by PtdOH in this way, such as the cyclic AMP phosphodiesterase PDE4A1 [4], Raf-1 kinase [5], sphingosine-1-kinase [6], phospholipase C isoforms [7], atypical protein kinase C isoforms [8], and p47-phox [9].

Identification of PtdOH-target proteins is often achieved by dissection of the pathways involving PLD, however, screening methods using a PtdOH-coated resin have indicated a number of interesting potential target proteins such as N-ethylmaleimide-sensitive factor (NSF), coatomer and ARF proteins [10]. Phage display is a well-established method for identifying novel proteins capable of interactions with a range of ligands, including proteins [11,12], lipids [13] and carbohydrates [14], so was used in the present study to identify potential PtdOH-target proteins. This highlighted a potential PtdOH-binding motif in the transmembrane solute transporter GLUT4.

GLUT4 translocation in response to insulin involves a number of lipid signalling molecules, including PtdIns(3,4,5)P_3_, PtdIns(3)P, and PtdIns(4,5)P_2_. These lipids play a major role in insulin signalling, as numerous studies have shown the PI3K inhibitor wortmannin to block insulin-stimulated increases in GLUT4 translocation and glucose uptake by over 90% [15-17]. The role of PLD-generated PtdOH in stimulation of glucose uptake is gradually being accepted. Some studies have demonstrated inhibition of GLUT4 translocation or glucose uptake with the inhibitor of PLD signalling, primary butanol [18,19], whereas another reported no such effect [20]. The work by Millar *et al *used a lower concentration of butanol and higher concentration of insulin than the other studies, thus it is possible that the stimulus was too great for the lower level of inhibitor to show an effect. Insulin appears capable of stimulating PLD activity [21-23], although the issue is complicated by the suggestion that the alcohol used to measure PLD activity may inhibit the insulin receptor [24]. PLD can be activated by ARF family proteins [25] and brefeldin A, which can inhibit the GTP loading of certain ARF proteins, inhibits insulin stimulation of PLD [21]. Increasing cellular levels of PLD protein by microinjection or viral transfection potentiates GLUT4 translocation in response to insulin [23,26]. Decreasing PLD1 levels with siRNA reduces GLUT4 exposure at the cell surface by affecting fusion, but not translocation or docking of the GLUT4-containing vesicles. Together these studies suggest an ill-defined role for PLD in the fusion of GLUT4-containing vesicles at the plasma membrane.

The phage display technique used in this study identified a motif present in GLUT4, but absent in other GLUT family members that are not thought to be regulated by PLD. Thus the potential PtdOH-binding motif was investigated for its involvement in insulin-stimulated GLUT4 translocation. We present here results showing that mutation of this motif impairs exposure of GLUT4 at the surface of 3T3-L1 adipocytes, via an effect on fusion of GLUT4-containing vesicles with the plasma membrane.

## Results

### Screening by phage display

**Figure 1:**
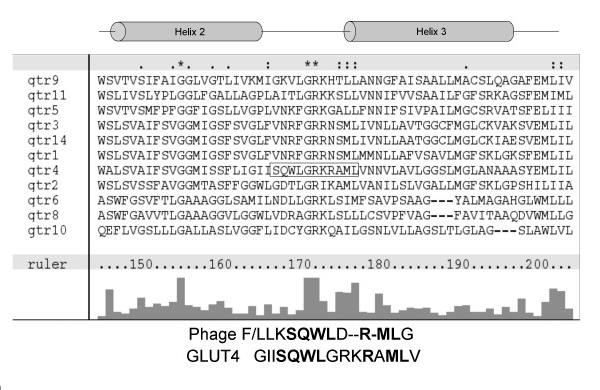
**ClustalX alignment of glucose transporter family sequences**. Amino acid sequences of the known glucose transporter family members were aligned using ClustalX. The potential PtdOH binding motif SQWLxRML (shown by boxed region) is only found in GLUT4, located in the cytoplasmic loop between helices two and three. Lower panel shows the alignment of the GLUT4 region with the phage display peptide, with homologous residues in bold type.

Potential phosphatidic acid (PtdOH)-binding motifs were identified by phage display using a randomised 12-mer phage library. Phage were panned over plastic and phosphatidylcholine-coated surfaces to remove non-specific hydrophobic surface-binding phage before incubation on the PtdOH-coated surface. Phage bound to the PtdOH surface were eluted with glycerol-3-phosphate which resembles the PtdOH headgroup to increase specificity of selection. Sequencing the resulting PtdOH-selected phage demonstrated a variety of 12-mer peptides consisting of predominantly hydrophobic and basic residues, in agreement with the characteristics of known PtdOH-binding sites such as Raf-1 kinase and p47-phox. The peptides were used as BLAST queries searching specifically for short nearly exact matches; this analysis returned a number of potential PtdOH-binding proteins. Notably, using the two resulting phage sequences FLKSQWLDRMLG and LLKSQWLDRMLG, the search identified the sequence SQWL-R – ML present in the solute transporter GLUT4, a sequence conserved in human, rat, mouse, pig and bovine genes. This was chosen for further study as GLUT4 translocation in response to insulin is believed to involve phospholipase D (PLD) signalling. The putative PtdOH-binding motif, SQWL, is located in the first intracellular loop of GLUT4, proximal to the third transmembrane helix and suitably placed to mediate interactions with a membrane lipid molecule. Notably, the other members of this solute transporter family, which are not thought to be regulated by PLD, do not contain this SQWL motif (Figure 1).

### Mutation of the SQWL motif permits translocation of GLUT4

**Figure 2:**
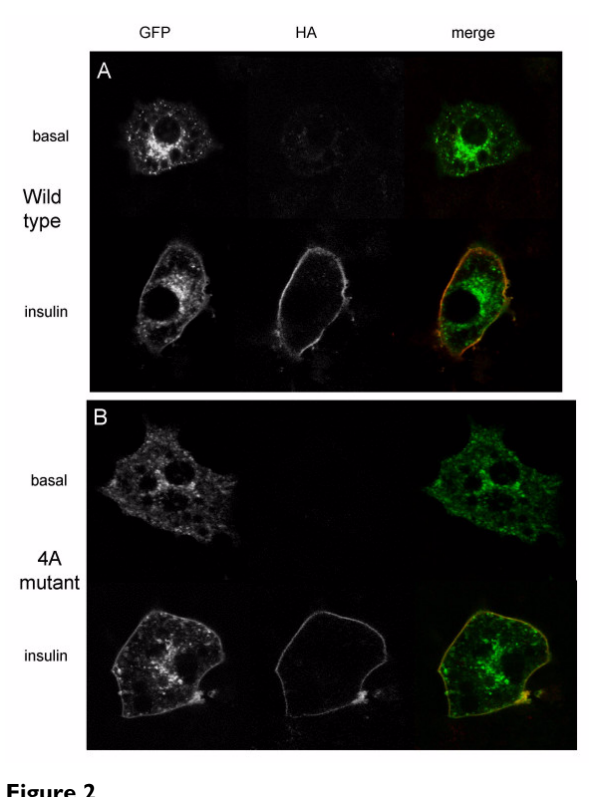
**Dual-tagged wild type and mutant GLUT4 translocates to the plasma membrane in response to insulin stimulation in 3T3-L1 adipocytes**. 3T3-L1 adipocytes were transiently transfected with (a) wild type or (b) 4A mutant GLUT4HA-GFP and serum starved for 4 hr, stimulated with 83 nM insulin for 10 min then fixed for confocal microscopy as described in Materials and Methods. Green staining shows total GLUT4HA-GFP, red denotes surface exposed HA epitope.

To allow assessment of GLUT4 translocation, GLUT4 containing an HA-tag in the first exofacial loop was tagged with GFP at the C-terminus. This allows cell surface-exposed GLUT4 to be measured relative to the total level of exogenous protein expressed, by comparing the ratio of HA to GFP in non-permeabilised cells. Figure 2a demonstrates that insulin stimulates the translocation of this double-tagged GLUT4 to the plasma membrane in transfected cells. 3T3-L1 adipocytes were also transfected with a dual-tagged GLUT4 in which the putative PtdOH-binding motif, SQWL, had been mutated to alanine residues; insulin was still able to stimulate translocation of this transporter, as assessed by confocal microscopy (Figure 2b). Similar data was obtained using transfected L6 myoblasts (not shown).

### Mutation of the SQWL motif reduces GLUT4 cell surface exposure in response to insulin

**Figure 3:**
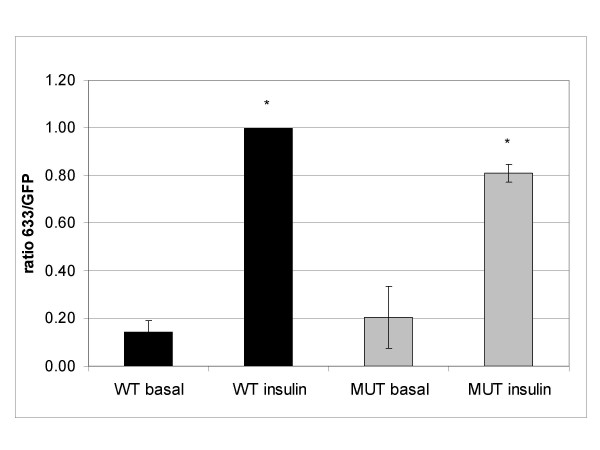
**Mutant GLUT4 shows less response to insulin than wild type GLUT4 in 3T3-L1 adipocytes**. 3T3-L1 adipocytes were transfected with wild type (WT, black bars) or 4A mutant (MUT, grey bars) pGLUT4HA-GFP and serum-starved for 4 hr, then stimulated with 83 nM insulin for 30 min. GLUT4 translocation was measured by quantitative confocal microscopy as described in Materials and Methods. Data are means of 3 independent experiments ± SEM. A ratio of 1.0 was set for insulin-stimulated wild type cells to normalise the data between the experiments. * indicates values are significantly different (ANOVA P < 0.0005).

To quantify the degree of GLUT4 exposure at the cell surface, we used the ratio of cell surface-exposed HA epitope signal to whole cell GFP signal. This effectively normalises the level of HA signal to the level of exogenous protein expressed in the cell, and therefore allows comparison of one cell with another. Quantification of the HA:GFP signal ratio from confocal microscope images of transfected adipocytes showed that wild type GLUT4HA-GFP demonstrated a five-fold increase in GLUT4 exposure at the cell surface in response to insulin. In contrast the mutated GLUT4 showed a decreased response to insulin, 20% ± 3% (mean ± SEM, ANOVA P < 0.0005) lower than for the wild type protein (Figure 3).

**Figure 4:**
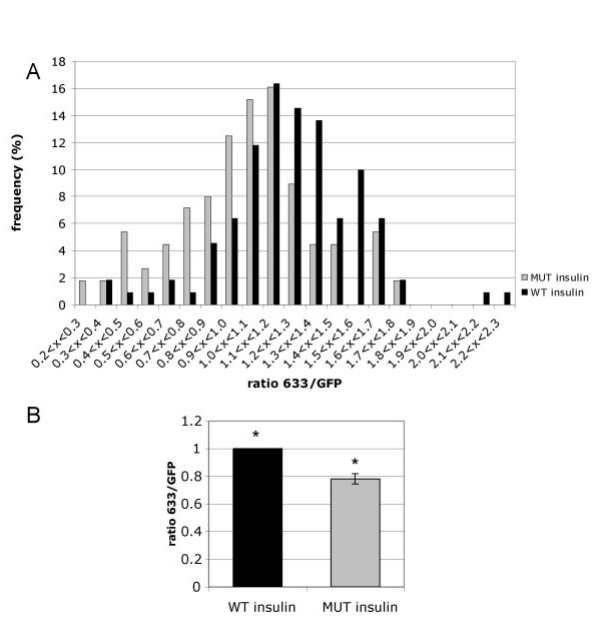
**4A mutation causes defect in fusion of GLUT4-containing vesicles at the plasma membrane**. 3T3-L1 adipocytes transfected with wild type (WT, black bars) or 4A mutant (MUT, grey bars) pGLUT4HA-GFP. Cells were serum-starved for 4 hr prior to stimulation with 83 nM insulin for 30 min. The ratio of Alexa633 to GFP signals at the plasma membrane was quantified as described in Materials and Methods. Histogram (a) shows distribution of ratios for wild type and mutant GLUT4-expressing cells expressed as a % of the total in each condition, and is representative of 3 independent experiments. Graph (b) shows means of 3 independent experiments ± SEM. The difference between wild type and mutant mean values is statistically significant (t-test P < 0.05, ANOVA P < 0.0005).

These images were then subjected to a more detailed analysis, in which the ratio of HA:GFP within a region of interest corresponding exclusively to the plasma membrane was measured. This provides an indication of the efficiency by which GLUT4 that has translocated to the plasma membrane is inserted into the membrane leaflet (i.e. fusion), and effectively normalises the HA signal to the level of GLUT4HA-GFP that has translocated to the membrane region to allow comparison between different cells. Analysis of the 4A mutant GLUT4 cells (Figure 4) showed that the mutation most likely affects fusion of GLUT4-containing vesicles at the cell surface, since the ratio of HA:GFP in the plasma membrane was lower than for the wild type protein (78% ± 3.7% mean ± SEM, normalised to wild type, t-test on means P < 0.05, ANOVA P < 0.0005). In contrast, the mutation had no significant effect on the insulin-stimulated translocation of GLUT4 to the plasma membrane region, as indicated by the ratio of plasma membrane GFP:whole cell GFP signal (1.21 ± 0.26 for wild type, mean ± SEM, 1.08 ± 0.28 for 4A mutant).

## Discussion

Insulin-induced glucose uptake via increased translocation of GLUT4 is believed to require PLD activity. Evidence for the role of PLD in insulin signalling has come from various methods, including treatment with the ARF inhibitor brefeldin A [21], microinjection or viral transfection of PLD [23,26], and PLD siRNA [23]. The siRNA study indicated a role for PLD specifically at the plasma membrane, regulating fusion of the GLUT4-containing vesicles at the cell surface.

There are several points at which PLD signalling could regulate GLUT4 cell surface expression. Firstly, PtdOH is a fusogenic lipid, promoting increased membrane curvature [27], so PtdOH generation may favour increased membrane curvature to allow fusion of GLUT4 vesicles to occur. Secondly, PtdOH may have a more defined role in signalling to regulate GLUT4 vesicle fusion. For example, fusion of GLUT4-containing vesicles with the plasma membrane involves formation of a *trans *SNARE complex between the v-SNARE VAMP2 and the t-SNAREs syntaxin-4 and SNAP23. which is later recycled by the proposed PtdOH-binding protein NSF. PLD-generated PtdOH could stimulate the ATPase activity of NSF to increase recycling of SNARE complex components and increase vesicle fusion. PtdOH also stimulates phosphatidylinositol 4-phosphate 5-kinase and atypical protein kinase Cζ activity, both enzymes involved in exocytosis [28-31]. As Unc13, the *C. Elegans *homologue of munc13 involved in exocytosis, binds diacylglycerol [32], increased phosphatidylinositol 4-phosphate 5-kinase activity could lead to increased DAG formation by phospholipase C and thereby regulate exocytosis via munc13. Additionally, overexpression of PKCζ potentiates insulin-stimulated GLUT4 translocation and glucose uptake via interaction with munc18c [33]. Munc18c binds syntaxin-4, inhibiting interactions between syntaxin-4 and VAMP2. This inhibition can be relieved by insulin-stimulated interaction of PKCζ with munc18c, enabling fusion to occur. Thus increased PtdOH levels could increase vesicle docking, tethering and fusion by a range of mechanisms. Here we have investigated a novel third hypothesis for regulation of GLUT4 translocation by PLD-generated PtdOH, a direct interaction of PtdOH with the GLUT4 protein.

Phage display, a well-established technique for identifying novel protein-ligand interaction motifs [11-14], highlighted a potential PtdOH-binding motif in the GLUT4 first intracellular loop. Mutation of this sequence reduced the insulin-induced exposure of GLUT4 at the cell surface of 3T3-L1 adipocytes by affecting fusion of the GLUT4-containing vesicles with the plasma membrane. It was not possible to directly experimentally measure PtdOH binding by GLUT4 protein, so an interaction between PtdOH and the SQWL motif *in vivo *cannot be definitively demonstrated. Since GLUT4 is a transmembrane protein, any additional specific protein-lipid interactions need not be sufficiently high affinity to enable detection of such interactions, as *in vivo *the proposed binding site would already be in close proximity to the lipid.

Inhibition of PLD activity by siRNA has been shown to reduce fusion of GLUT4 vesicles. This can be rescued by addition of lyso-phosphatidylcholine, another fusogenic lipid. Simply favouring membrane curvature by the addition of fusogenic lipids may be sufficient to overcome the energy barrier associated with vesicle fusion in an experimental system, but more specific mechanisms may function *in vivo*. The current work suggests that fusion of GLUT4-contining vesicles with the plasma membrane is facilitated by a change in GLUT4 conformation upon binding to PtdOH, such that GLUT4 is better recognised by other regulators of vesicle fusion and/or allowing adoption of increased membrane curvature necessary for the formation of the fusion pore intermediate. The conformation of membrane-associated proteins has previously been shown to regulate membrane curvature, e.g. the BAR domain of endophilin stabilises membrane curvature via a number of electrostatic interactions [34], and the GTPase Sar1p promotes membrane curvature by insertion of an amphipathic α-helix into the membrane [35].

## Conclusion

The data presented here demonstrate the identification of a potential PtdOH-binding site by phage display, in the first intracellular loop of GLUT4. Mutation of this site is capable of modulating the efficiency of GLUT4-containing vesicle fusion. It remains to be shown how this motif regulates fusion, whether by favouring membrane curvature or recognition of the vesicle by other trafficking regulators. This region is unique to GLUT4 among the sugar transporters and thus may point to a unique mechanism of regulation involving lipid signalling.

## Methods

### Materials

Lipids egg phosphatidylcholine (PtdCho), and palmitoyl oleoyl phosphatidic acid (PtdOH) were from Avanti Polar Lipids (Alabama, U.S.A). Tetramethylbenzidine (TMB) super sensitive peroxidase substrate was from Tebu-bio (Peterborough, U.K). Fatty acid-free bovine serum albumin was from Sigma (Gillingham, U.K). Mouse monoclonal anti-HA antibody was from Covance (Cambridge Antibody Technologies, Cambridge, U.K.). Cell culture medium and Alexa-tagged secondary antibodies were from Invitrogen (Paisley, U.K).

### Screening by phage display

96-well dilution plate wells (Falcon) were coated with 200 pmoles per well egg phosphatidylcholine or palmitoyl oleoyl phosphatidic acid in hexane/ethanol 1:1. Plates were dried in air for 1 hr, then under vacuum for 1 hr. Lipid-coated and uncoated wells were blocked with blocking buffer (0.1 M NaHCO_3_, 5 mg/ml BSA, 0.02% NaN_3_, 2.5 μM CaCl_2 _pH8.6) at 4°C overnight, then the wells washed six times with TBST (50 mM Tris.HCl pH 7.5, 150 mM NaCl, 0.1% Tween20, 2.5 μM CaCl_2_). An M13 12-mer phage library (New England Biolabs) diluted in TBST was panned sequentially over plastic (non-coated wells), egg PtdCho then PtdOH surfaces. Phage not bound to the PtdOH surface were washed away with TBST, and bound phage were eluted with phage elution buffer (0.5 mM glycerol-3-phosphate, 50 mM Tris.HCl pH 7.5, 150 mM NaCl, 0.1% Tween20, 2.5 μM CaCl_2_). Panning was repeated for second and third rounds, after which PtdOH-specific phage were isolated and amplified according to the manufacturer's protocol. The phage DNAs encoding the 12-mer peptides were sequenced and the resulting sequences entered as BLAST database queries.

### Constructs

pCis-GLUT4HA (HA-tag in first exofacial loop) was obtained from Prof. G. Gould. The GLUT4HA open reading frame was cloned by PCR into pEGFPn2 to produce pGLUT4HA-GFP, and verified by sequencing. Site-directed mutagenesis of Ser103-Leu106 to alanines (Stratagene, Quikchange kit) was carried out according to the manufacturer's protocol.

### Cell culture and transfection

3T3-L1 fibroblasts were cultured in Dulbecco's Modified Eagle Medium (DMEM) supplemented with 10% foetal calf serum and penicillin/streptomycin. Cells were passaged every 3 days to maintain 30–90% confluence. Differentiation into adipocytes was carried out as described previously [36]. 3T3-L1 adipocytes were transfected by electroporation, 90 μg DNA per 0.4 cm cuvette (180 V, 950 μF) then seeded in coverslip-bottomed dishes.

### Confocal microscopy GLUT4 translocation assay

Transfected 3T3-L1 adipocytes on collagen-coated glass coverslips (Mattek) were serum-starved for 4 hr prior to stimulation with 83 nM insulin for 30 min at 37°C. All further steps were carried out at room temperature. After stimulation, cells were fixed with 4% paraformaldehyde for 20 min. Coverslips were washed three times with phosphate buffered saline (PBS), and blocked with 3% bovine serum albumin (BSA) in PBS for 45 min. Primary antibody mouse monoclonal anti-HA (Covance) was incubated at 1:500 in blocking buffer for 45 min. After washing three times in PBS, secondary antibody Alexa 633-conjugated anti-mouse IgG (Invitrogen) was used at 1:500 in blocking buffer and incubated for 45 min. Slides were washed three times in PBS and left in PBS in the dark at 4°C ready for microscope analysis.

GFP-expressing cells were imaged using a Leica DM IRBE system with 488 nm excitation for GFP and 633 nm excitation for the Alexa 633 secondary antibody. Emission filters were set at 499–551 nm for GFP and 649–682 for Alexa 633. PMT settings were kept constant during individual assays to allow quantification. At least 35 cells were imaged from each condition, and the images analysed using Metamorph software (Molecular Devices). Transfected cells were selected, and the average pixel intensity for both GFP and 633 channels calculated. These values were used to calculate a ratio of the proportion of GLUT4HA-GFP exposed at the plasma membrane relative to the total level of protein expressed. Further analysis of the GFP:633 ratio was carried out by selecting the plasma membrane of the cell as the region of interest; this allows a determination of the efficiency of GLUT4 insertion (fusion) into the plasma membrane to be undertaken. Pixel intensity values were corrected for background of non-transfected cells, and normalised to the wild type GLUT4 insulin response data. Insulin-induced translocation from the intracellular storage compartment to the plasma membrane region was assessed by the ratio of plasma membrane GFP: whole cell GFP signals. 13 to 31 cells were analysed for each condition, and the mean ratios calculated. Statistical analysis used the Student's T test and ANOVA on SPSS 13.0 software.

## Authors' contributions

CAH carried out the molecular biology, microscopy and image analysis, participated in the sequence alignment and drafted the manuscript. TRP carried out the phage display and participated in the sequence alignment. SEL, GIW and JMT participated in the design and coordination of microscopy experiments, and helped draft the manuscript. SEL also participated in carrying out the microscope work. MJOW supervised the design and coordination of all experiments and helped draft the manuscript.
